# Colorectal cancer with testicular metastasis: A case report and literature review

**DOI:** 10.1097/MD.0000000000033214

**Published:** 2023-03-17

**Authors:** Jia-Ming Wu, Ao Zhang, Yu Dong, Si-Hong Lin, Jin-Cheng Meng, Can-Tu Fang

**Affiliations:** a Zhongshan Hospital of Traditional Chinese Medicine Affiliated to Guangzhou University of Chinese Medicine, Zhongshan, China; b Clinical Research and Big Data Laboratory, South China Research Center for Acupuncture and Moxibustion, Medical College of Acu-Moxi and Rehabilitation, Guangzhou University of Chinese Medicine, Guangzhou, China.

**Keywords:** case report, chemotherapy, intestinal cancer, metastasis mechanism, testicular metastasis

## Abstract

Colorectal cancer is the second leading cause of cancer-related deaths in the world. About 50% of patients will have metastases during the course of the disease. The common sites of metastasis are the liver, lung, peritoneum, lymph, etc. Metastatic carcinoma to the testes is uncommon. We found a case of ascending colon cancer metastasized to the testis in the clinic.

**Patient concerns:**

We reported a 50-year-old male patient who was found to have testicular metastases >4 years after intestinal cancer surgery, and multiple metastases in the peritoneum and pelvis were found 1 week later.

**Diagnoses:**

After enhanced computed tomography and pathological biopsy, the patient was diagnosed with testicular metastasis of colon cancer.

**Interventions:**

Capecitabine combined with bevacizumab is currently undergoing palliative treatment.

**Outcomes:**

The patients died of tumor progression on June 28, 2021.

**Lessons:**

The testicular metastasis of colorectal cancer is a sign of peritoneum and multiple metastases. When the testicular metastasis occurs in colorectal cancer patients, it usually indicates that the patient has a poor prognosis.

## 1. Introduction

Colorectal cancer accounts for 10.2% of all cancers worldwide, the third highest incidence but the second highest mortality rate.^[1]^ Studies have shown that more than half of patients with colorectal cancer will develop distant metastasis after surgery. The 5-year survival rate for stage I colon cancer is 90%, while the 5-year survival rate for stage IV colorectal cancer with distant metastasis is only 14%. Therefore, metastasis is the main cause of treatment failure of colorectal cancer.^[2]^ The common metastasis route of colorectal cancer is liver (60%),^[3]^ followed by lung (10–15%),^[4]^ including peritoneum, bone, brain, kidney, etc while testicular metastasis of colorectal cancer is extremely rare.^[5]^ Recently, a case of colorectal cancer with testicular metastasis was discovered in our hospital, which is reported as follows.

## 2. Case presentation

**Figure 1.**
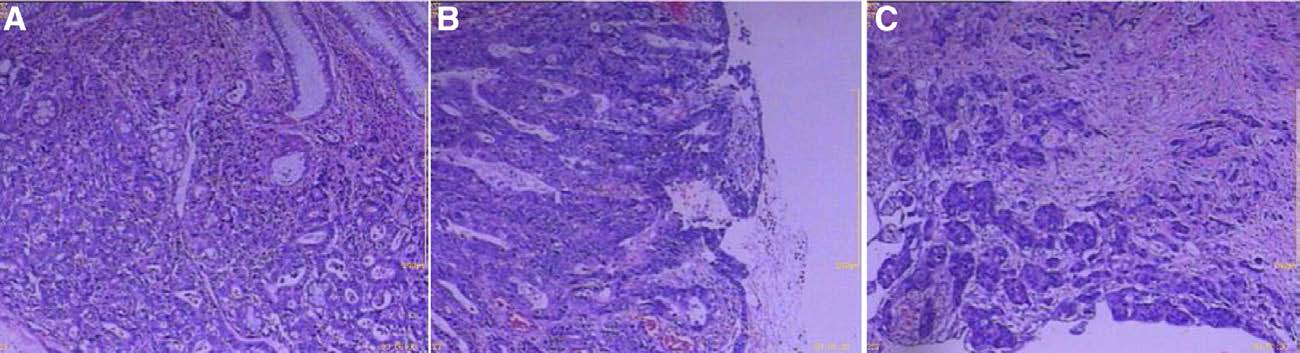
Pathology and immunohistochemistry of ascending colon tumor.

In August 2016, a 50-year-old Chinese male patient developed blood in stool with pain in the right lower abdomen and abdominal distension without any obvious causes. He has lost 6 kg in weight since the onset of the disease. Physical examination showed no abnormality, and no history of malignant tumor was found in his known relatives. On January 4, 2017, laparoscopic-assisted radical resection of ascending colon cancer was performed under general anesthesia. During the operation, a 3 × 4 cm mass with hepatic curvature of ascending colon was observed, invading the whole layer of the intestinal wall. Postoperative pathology showed that (Fig. 1) moderately to poorly differentiated adenocarcinoma penetrated the muscular layer and reached the parenteral tissue, with vascular invasion and no invasion of nerve bundles. Cancer cells were found in 7 of 29 lymphaden. Immunohistochemical results: local CK7 (+), local CK20 (+), GATA-3 (−), P53 (2+, about 70%), Ki-67 (+, about 60%), CDX-2 (+), Villin (+). Tumor staging: T3N2M0 III stage C.

**Figure 2.**
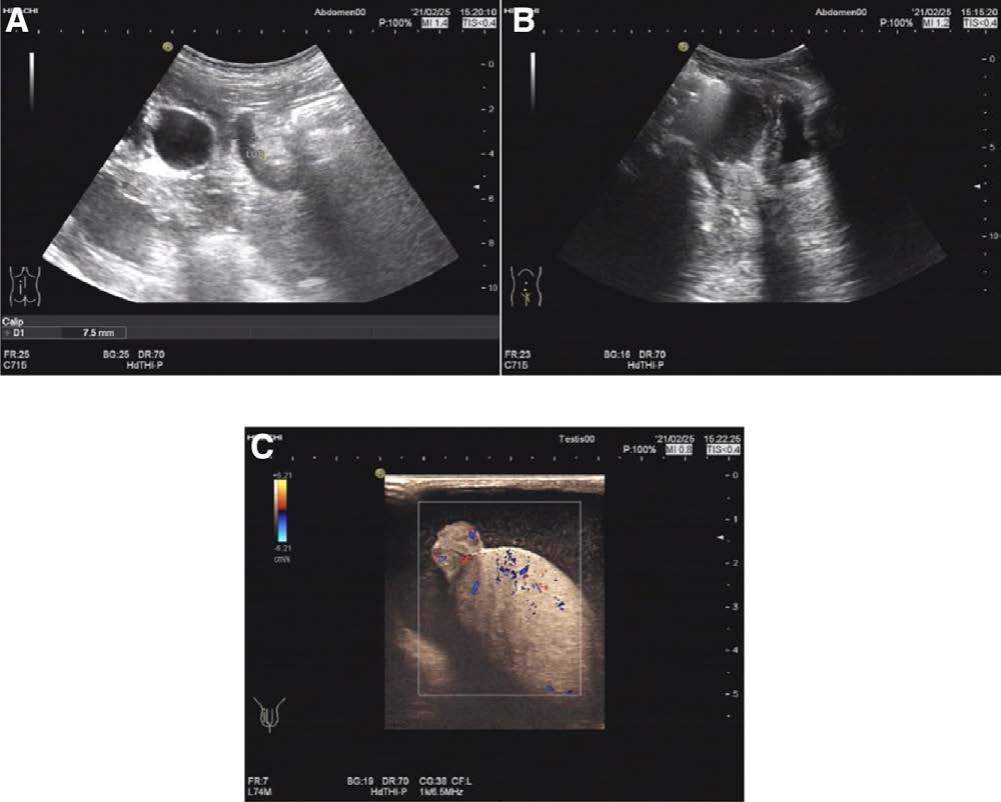
Color ultrasonography of scrotum.

**Figure 3.**
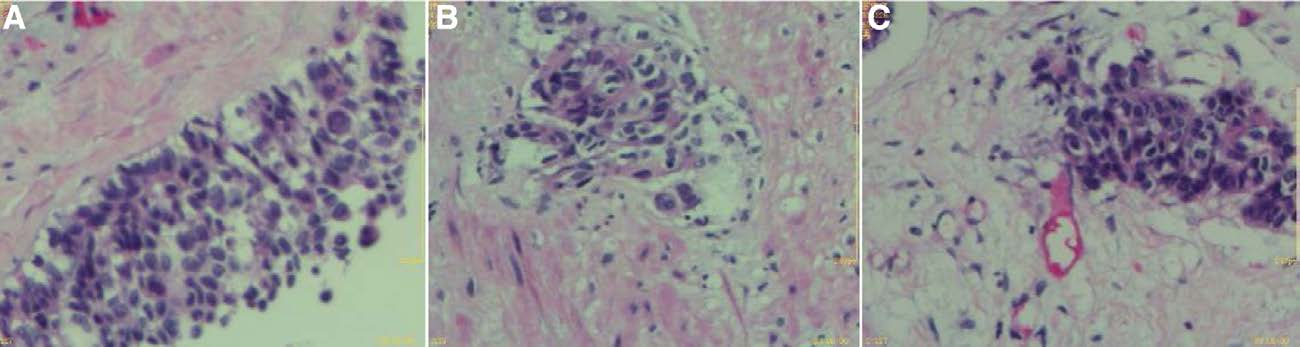
Pathology of testicular mass.

**Figure 4.**
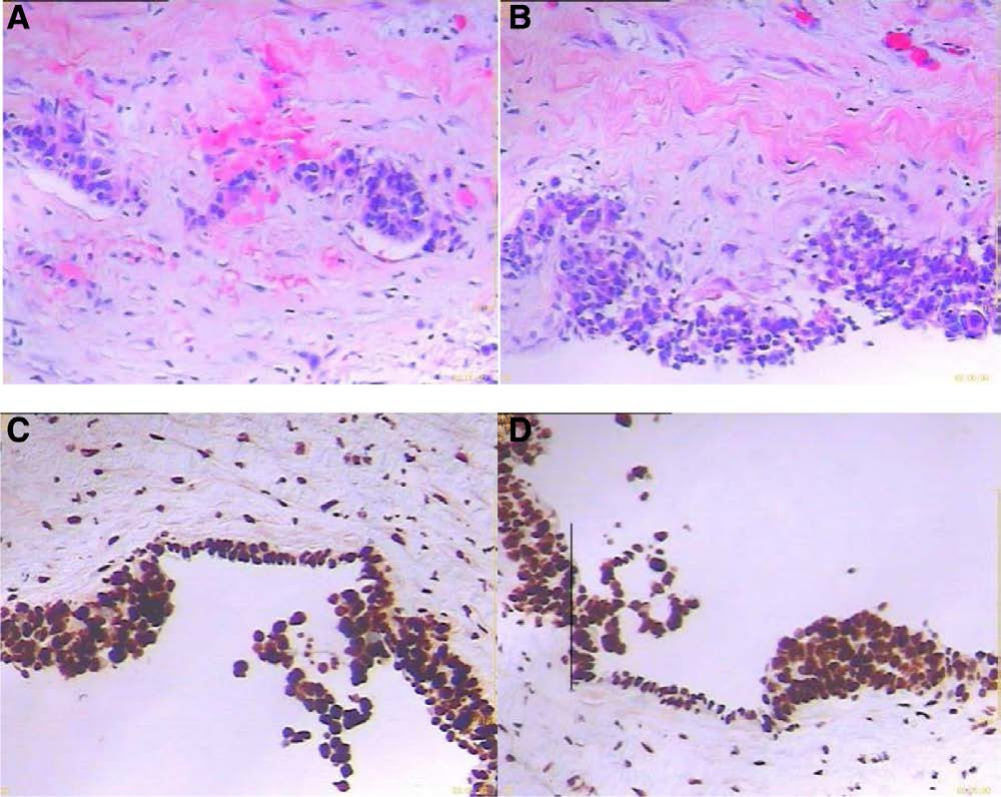
Immunohistochemistry of testicular masse.

After surgery, 8 courses of chemotherapy were administered with XELOX (oxaliplatin 200 mg vd d1, capecitabine 1500 mg Po bid d1-14), The last time of chemotherapy was on July 25, 2017. Regular reexamination was conducted after chemotherapy. On February 25, 2021, the patient was readmitted due to right scrotal mass found >6 months later. Color ultrasonography of the scrotum indicated hydrocele of the right testis sheath (Fig. 2), carcinoem-bryonic antigen, human chorionic gonadotropin β, human epididymal protein and carbohydrate antigen 125 were normal. On physical examination, a mass of about 4.0 × 4.0 cm was found in the right scrotum, which cannot be reduced when lying flat, and there is no obvious pain or discomfort. Therefore, on February 26, 2021, the right testicular sheath reversal resection was performed. Postoperative immunohistochemical findings: MSH6 (+), MSH2 (+), MLH1 (+), PMS2 (+). Pathological findings: (right testicular sheath, Figs. 3 and 4) combined with history, immunohistochemistry, and morphological changes were consistent with poorly differentiated adenocarcinoma (of gastrointestinal origin) with hydrocele.

**Figure 5.**
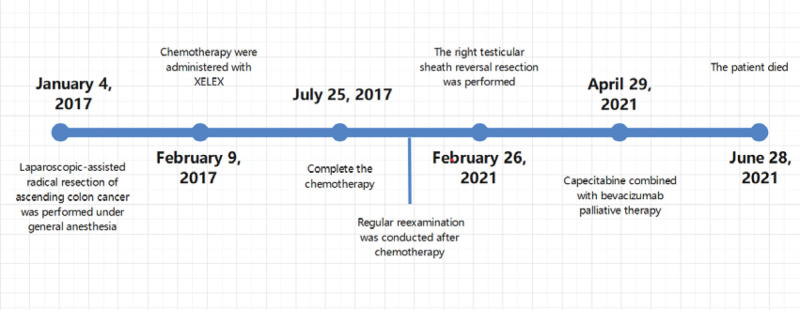
Timeline.

Genetic test: negative for BRAF (V600E in the outside 15) mutation, negative for KRAS and NRAS gene mutation. One week after surgery, enhanced abdominal computed tomography reexamination revealed new thickening and enhancement of right peritoneum, right retroperitoneal cavity, and pelvic cavity, and metastasis was considered. New metastatic tumor of right kidney and high-density nodules of L3 vertebral body were considered for metastasis. Tumor staging: rTxNxM1c stage IV. Because the patient could not tolerate high-intensity chemotherapy, he was only given capecitabine combined with bevacizumab for palliative treatment and died on June 28, 2021 (Fig. 5).

## 3. Discussion

Secondary tumors of the testis accounted for only 1.0%.^[5]^ The mechanism of testicular metastasis is unclear. It is believed that the occurrence of testicular metastasis depends on multiple factors. Some scholars speculate that the low-temperature environment of the testis may affect tumor growth.^[6]^ Some scholars believe that the adequate closure of the inguinal ring blocks the metastasis of the tumor,^[7]^ that this may be related to the congenital blood-testicular barrier in the testis. Testicular metastases were most common in patients with prostate cancer (35%), followed by lung cancer (18%), melanoma (18%), kidney cancer (9%), etc. Testicular metastasis (<8%) from colorectal cancer is extremely rare.^[5]^

In Table 1, we collected 21 articles on colorectal cancer metastases to the testis and found that the reported patients with testicular metastases from colorectal cancer ranged from 15 years old to 77 years old. The average age is about 52 to 53, which is higher than the age of patients with primary testicular tumors and is similar to the average age of 51 years old reported in other literature for patients with secondary testicular tumors.^[23]^

**Table 1:** Clinical feature of previously reported.

	Author	Year report	Age	Primary lesion site	Pathological typing	Clinical symptoms of testicular metastasis	Time from testicular metastasis to detection of bowel cancer
1	Belsky and Konwaler^[8]^	1954	25	Transverse colon	Not noted	No genital symptoms	Autopsy found
2	Cricco and Kandzari^[9]^	1977	47	Cecum	Mucinous adenocarcinoma	Testicular swelling	Simultaneous discovery
3	Jubelirer^[10]^	1986	52	Sigmoid flexure	Moderately differentiated adenocarcinoma	Testicular tumors	1 yr and 11 mo
4	Randall et al^[11]^	1988	32	Descending colon	Moderately differentiated adenocarcinoma	Pain and swelling of testicles	Testicular metastasis is the first sign
5	Bryan et al^[12]^	1997	75	Sigmoid flexure	Adenocarcinoma	Scrotal swelling	Simultaneous discovery
6	Nello et al^[13]^	2004	62	Not noted	Adenocarcinoma	No genital symptoms	Simultaneous discovery
7	Tiong et al^[14]^	2005	76	Sigmoid flexure	Moderately differentiated adenocarcinoma	Increase testicular	Testicular metastasis is the first sign
8	Charles et al^[15]^	2005	40	Sigmoid flexure	Poorly differentiated adenocarcinoma	Pain in the left groin	11 mo
9	Hatoum et al^[16]^	2006	65	Rectum	Moderately differentiated adenocarcinoma	Right testis enlargement	6 yr and 2 mo
10	Ouelle- -tte et al^[17]^	2007	51	Rectum	Adenocarcinoma	Enlarged right testicle	9 mo
11	Jesús Martínez Ruiz et al^[18]^	2010	24	Cecum	Mucinous adenocarcinoma	Severe testicular pain	10 mo
12	Ramachandran et al^[19]^	2010	71	Rectum	Moderately differentiated adenocarcinoma	Nodule in the right testis	5 yr
13	Badereddin^[6]^	2012	77	Descending colon	Adenocarcinoma	Pain in left inguinal and testicular	2 yr and 6 mo
14	Rampa et al^[20]^	2012	Not noted	Sigmoid colon	Not noted	Painless testicular nodule	3 yr
15	Verma et al^[21]^	2013	35	Rectum	Mucinous adenocarcinoma	Testicular mass and ascitis	Testicular metastasis is the first sign
16	Qi Xu et al^[7]^	2015	73	Descending colon	moderately differentiated adenocarcinoma	Right inguinal and left Abdominal pain	Testicular metastasis is the first sign
17	Foster et al^[22]^	2016	52	Rectum	Moderately differentiated adenocarcinoma	Testicular tumors	1 yr and 4 mo
18	Omar et al^[23]^	2016	43	Cecum	Adenocarcinoma	Dull right groin and scrotal pain	Testicular metastasis is the first sign
19	Singh et al^[24]^	2018	15	Rectum	Signet ring cell adenocarcinoma	Right testicular nodule	At the same time
20	Smit et al^[25]^	2019	75	Sigmoid flexure	Adenocarcinoma	Swelling and painful right testicle	1 yr
21	Gabsi et al^[5]^	2021	37	Rectum	Moderately differentiated adenocarcinoma	Right hydrocele with hetero nodular testis	At the same time

Most testicular metastases are found at the same time as intestinal cancer, and even testicular metastasis is the first symptom. Testicular metastasis is more likely to occur within 2 years after the discovery of intestinal cancer, and rarely >5 years. Metastatic carcinoma of the testis is more common on 1 side only. Robert^[26]^ reported a patient with bilateral testicular metastasis from colorectal cancer. The most common testicular metastasis of colorectal cancer is rectal cancer, followed by sigmoid colon cancer, cecum cancer, descending colon cancer, and transverse colon cancer. No reports of ascending colon cancer testicular metastasis have been found yet. Moderate to poorly differentiated adenocarcinoma is most prone to testicular metastasis, and the most common pathological type of testicular metastasis is mucinous adenocarcinoma.

Most patients with testicular metastatic bowel cancer present with testicular swelling or pain, and a few present with hydrocele. Therefore, clinically elderly patients with testicular swelling or hydrocele, together with other system symptoms or medical history, should be considered for the possibility of metastatic cancer to the testis. There are still many patients who have no symptoms and are only found during physical examination. Therefore, for male patients with colon cancer who are at a high incidence of testicular metastasis, testicular examination can be used as a routine physical examination during follow-up diagnosis to find the lesion as soon as possible and treat it as soon as possible.

The metastasis of colorectal cancer includes arterial embolization, retrograde lymphatic diffusion, retrograde venous diffusion, and direct invasion of surrounding tissues.^[27]^ Once testicular metastasis occurs in colorectal cancer, the prognosis is very poor. Literature has shown that the average survival time after diagnosis of testicular metastasis is only 6 to 12 months.^[21]^ The presence of testicular metastasis has been suggested as a possible marker of peritoneal metastasis.^[25]^ Therefore, testicular metastasis of colorectal cancer should be vigilant in clinical practice.

## Acknowledgment

The authors would like to thank the patient, who consented to have her data published in this case report.

## Author contributions

**Data curation:** Si-Hong Lin.

**Supervision:** Jin-Cheng Meng, Can-Tu Fang.

**Writing – original draft:** Jia-Ming Wu, Ao Zhang.

**Writing – review & editing:** Yu Dong.
